# Feasibility of mechanomyography-based fatigue classification for passive lower-limb exoskeleton evaluation: A pilot study

**DOI:** 10.1371/journal.pone.0350941

**Published:** 2026-06-10

**Authors:** Sijing Wang, Xiaorong Guan, Huibing Li, Rui Zhang, Yu Bai, Qiang Zhou

**Affiliations:** 1 School of Mechanical Engineering, Nanjing University of Science and Technology, Nanjing, China; 2 Chongqing Jianshe Industry (Group) Co., Ltd, Chongqing, China; 3 Air Force Hospital of Eastern Theater Command, Nanjing, China; Indian Institute of Technology Patna, INDIA

## Abstract

Mechanomyography (MMG) enables non-invasive monitoring of muscle mechanical activity, while its utility in time-resolved fatigue detection during dynamic human–exoskeleton interaction remains underexplored. This pilot study explored the feasibility of combining MMG with machine learning to characterize neuromuscular fatigue and evaluate passive lower-limb exoskeleton assistance during repetitive 10 kg squat-lifting tasks. MMG signals from five lower-limb muscles were extracted for time-domain, frequency-domain and nonlinear features, and fatigue identification was implemented via a spectral-based criterion and multi-muscle voting optimization. A radial basis function-enhanced random forest (RBF-RF) model integrated with data augmentation was validated through leave-one-subject-out cross-validation. The results demonstrated that the 1/5 voting rule achieved optimal performance, with mean accuracy of 0.913 ± 0.057, AUC of 0.792 ± 0.073, and a low fatigue detection error of 1.4 ± 0.8 s. Data augmentation steadily improved model robustness, and predicted fatigue levels were significantly correlated with subjective perceived exertion (ρ = 0.756, p < 0.001). This pilot study demonstrates the feasibility of MMG-based fatigue monitoring for wearable assistive systems. The proposed framework supports objective, high-temporal-resolution fatigue monitoring, and may serve as a viable tool for assessing wearable assistive systems. Further large-cohort studies are required to validate its generalizability for practical applications.

## 1. Introduction

Work-related musculoskeletal disorders (WMSDs) pose a critical global challenge, particularly in manual labor sectors like construction, manufacturing, and logistics, stemming from cumulative fatigue from repetitive motions, heavy lifting, and awkward postures [1]. In the European Union, approximately 30% of workers are engaged in manual material handling, 63% perform repetitive movements, and 46% are exposed to awkward postures—figures that have remained largely unchanged over the past decade [2]. Correspondingly, more than 40% of workers report experiencing low back or neck/shoulder pain annually [3]. These risks are particularly pronounced in construction-related occupations, where high-frequency lifting tasks and heavy loads substantially elevate WMSD incidence and contribute to premature workforce exit [4,5]. These challenges motivate the development of wearable interventions and, critically, the need for practical methods to evaluate their effectiveness at the muscular level.

Wearable exoskeletons have been proposed as a promising intervention to mitigate such physical burdens by redistributing loads and reducing musculoskeletal fatigue [6]. Both active (motor-driven) and passive (mechanical) exoskeletons have demonstrated potential benefits in clinical rehabilitation, including mobility assistance for individuals with neurological impairments [7–10], as well as in industrial settings, where reductions in joint loading and metabolic cost during lifting and walking tasks have been reported [11–13]. Despite these encouraging findings, the objective evaluation of exoskeleton efficacy—particularly under dynamic, real-world working conditions—remains a significant challenge [1,12].

A central obstacle lies in the lack of robust and practical evaluation metrics [14]. Existing assessment approaches can be broadly categorized into work performance evaluation [9], biomechanical evaluation [15],metabolic cost evaluation, and physiological bio-signal evaluation. Operational performance evaluation, while useful for demonstrating task compatibility [7,9,10] is inherently task-specific, which severely limits its generalizability across diverse functional scenarios. Kinematic and dynamic parameter assessments offer precise insights into joint mechanics and torque reduction [16], yet their reliance on expensive 3D motion-capture environments renders them impractical for ecological, real-world applications. Although metabolic cost evaluation provides a measure of energetic efficiency [11,13], the approach is hampered by prolonged data collection requirements, respiratory noise, and the physical burden of the metabolic equipment itself. While each provides valuable insights, they are often limited by task specificity, high equipment cost, lengthy protocols, or poor suitability for field deployment. Among these, bio-signal-based fatigue assessment is widely regarded as a direct and physiologically meaningful indicator of musculoskeletal load and exoskeleton assistance [17]. Bio-signal assessment is dominated by surface electromyography (sEMG), while electroencephalographic (EEG) applications remain limited in ambulatory settings [18]. However, sEMG is highly sensitive to practical challenges in actual industrial fields: skin impedance variations, sweat-induced signal degradation, electrode displacement or detachment, motion artifacts, and electromagnetic interference. These factors significantly compromise signal reliability during dynamic industrial tasks (e.g., repetitive lifting, material handling), limiting its real-world applicability [19].

Subjective fatigue scales, such as the Borg Rating of Perceived Exertion, are frequently employed as complementary tools but exhibit substantial inter-individual variability and limited reproducibility, restricting their utility as standalone evaluation criteria [20,21]. Consequently, there is a growing need for alternative physiological signals that are more robust to environmental and motion-related disturbances while remaining practical for use with wearable exoskeleton systems.

Unlike sEMG, mechanomyography (MMG) provides a robust, practical alternative for monitoring muscle activity and fatigue in dynamic occupational settings. MMG captures low-frequency mechanical oscillations from muscle fiber contractions [22], and direct comparative studies have validated its quantitative superiority: MMG yields a 14-fold higher signal amplitude (~2.8 V) and 7-fold greater signal-to-noise ratio (SNR ~ 25) compared to sEMG (~0.2 V amplitude, SNR ~ 4) under identical dynamic conditions [23]. MMG is inherently resilient to skin impedance changes, sweat, or dirt [24], supports signal acquisition through clothing without skin preparation (e.g., shaving, cleaning, or gel application), and maintains higher sensitivity to load variations than sEMG during dynamic lifting tasks [25]. This enables quick, hassle-free sensor placement and superior flexibility for integration into wearable exoskeletons [26], while MMG features such as RMS amplitude and frequency-domain indices remain highly sensitive to activation and fatigue [27].

Nevertheless, MMG-based fatigue assessment faces methodological challenges, including non-stationary signals prone to motion artifacts and external vibrations in industrial environments [28], absence of standardized preprocessing pipelines, and obscured spectral shifts during dynamic tasks [29]. Although machine-learning classifications [27,30], and sEMG-MMG fusion can enhance accuracy [31], MMG-only approaches offer greater real-world potential for exoskeleton-assisted lifting due to their minimal setup requirements, tolerance to harsh conditions, enhanced user acceptance and compliance, and lower system complexity and cost—advantages that prioritize seamless integration into demanding industrial workflows.

Against this background, the present study aims to explore the feasibility of an MMG-based fatigue evaluation framework for assessing the fatigue-delaying efficacy of a passive lifting exoskeleton. Specifically, we propose an end-to-end framework combining optimized MMG signal preprocessing with a machine learning-based fatigue classification model, and we conduct a pilot experimental study (N = 5) involving repetitive squat-lifting tasks with and without exoskeleton assistance. Rather than asserting definitive performance improvements of the exoskeleton, and consistent with the exploratory focus of this work, this study seeks to provide initial evidence on the practicality, robustness, and potential value of MMG-only fatigue assessment in exoskeleton evaluation, thereby laying the groundwork for larger-scale validation studies. Accordingly, the emphasis is placed on methodological feasibility and sensitivity rather than on definitive efficacy claims.

## 2. Materials and methods

### 2.1. Participants

Five healthy adults (*N* = 5; 5 males; age: 24 ± 3 years; height: 174.5 ± 6.5 cm; weight: 71.5 ± 11.5 kg) were recruited for this pilot feasibility study. Participant recruitment was conducted between 28/12/2024 and 29/12/2024. All participants were right-leg dominant and met the following inclusion criteria: (1) no history of lower-limb musculoskeletal or neurological disorders; (2) no history of smoking or alcohol abuse; and (3) agreement to refrain from strenuous physical activity for 48 hours prior to testing to eliminate pre-existing muscle fatigue.

The small sample size aligns with the exploratory nature of this pilot design, which focuses on validating the feasibility of the MMG-based fatigue evaluation framework for exoskeleton efficacy assessment rather than drawing definitive population-level inferences. The study protocol was approved by the Medical Ethics Committee of Nanjing Qixia District Hospital (Approval No.: 2024-QX029) and conducted in strict accordance with the Declaration of Helsinki. All participants provided written informed consent prior to study participation.

### 2.2. Experimental apparatus and procedures

**Fig 1:**
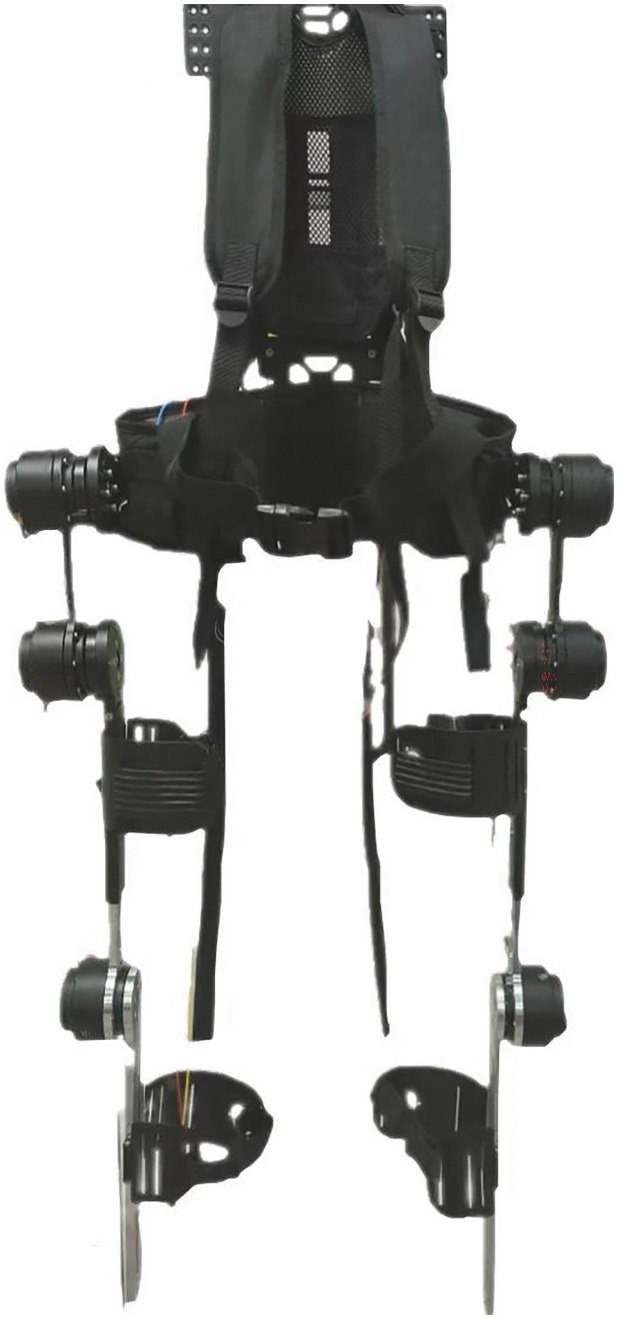
A passive lower-limb exoskeleton.

A custom-designed passive lower-limb exoskeleton was used for the experiment (Fig 1). This exoskeleton employs a spring-based mechanical mechanism to deliver supportive torque during hip and knee extension, engineered to reduce metabolic and muscular loads on the lower back and lower-limb muscles during repetitive squat-lifting. It features adjustable waist and thigh components to accommodate participants with heights ranging from 165 cm to 182 cm, with articulations aligned to human biomechanics to prevent restrictions on natural movements including squatting and standing.

**Fig 2:**
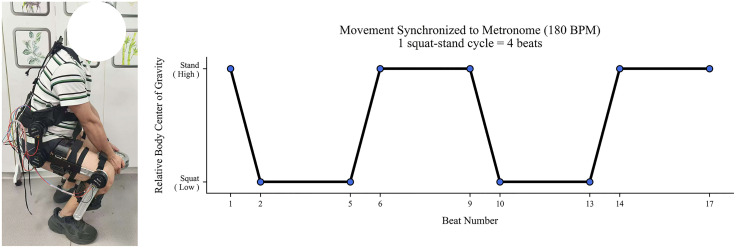
Schematic of the repetitive squat-lifting task and metronome pacing.

Each participant completed two experimental conditions in randomized order on the same test day: unassisted (no device) and exoskeleton-assisted repetitive squat-lifting. Prior to trials, all participants completed a 5-minute standardized warm-up (bodyweight squats and static leg stretches) to reduce injury risk. A 30-minute rest period was provided between conditions to minimize residual fatigue. For both conditions, participants performed repetitive squat-lifting of a 10 kg load, synchronized to a digital metronome (180 beats per minute; one squat-stand cycle = four beats) to standardize movement pace as illustrated in Fig 2.

To obtain a secondary measure of perceived exertion for convergent validity testing, participants verbally reported their fatigue level every 5 seconds using a 6-point ordinal scale (0–5), as detailed in Table 1. This scale is adapted from the classic Borg Rating of Perceived Exertion (RPE) 0–10 [32]. The simplified 6-point structure is designed to minimize the cognitive burden of real-time fatigue reporting during dynamic repetitive squat-lifting while retaining the core perceptual gradient—ranging from “no feeling” (0) to “exhaustion” (5). To align with the binary fatigue states defined by MMG (Class A: non-fatigued; Class B: fatigued), scores of 0–2 were mapped to Class A and scores of 3–5 to Class B. This threshold reflects the commonly accepted transition from moderate to high perceived exertion, reflecting the transition from mild discomfort to significant fatigue. All trials were terminated at voluntary exhaustion (inability to maintain the metronome rhythm for three consecutive cycles), ensuring capture of the complete muscular load trajectory prior to physical failure.

**Table 1:** Simplified Rating of Perceived Exertion (RPE) Scale.

Subjective Feeling	Subjective Exertion Scores
**No feeling**	0
**Slightly strenuous**	1
**Strenuous**	2
**Very strenuous**	3
**Extremely strenuous**	4
**Try your best**	5

### 2.3. MMG data acquisition, preprocessing, and fatigue state definition

**Fig 3:**
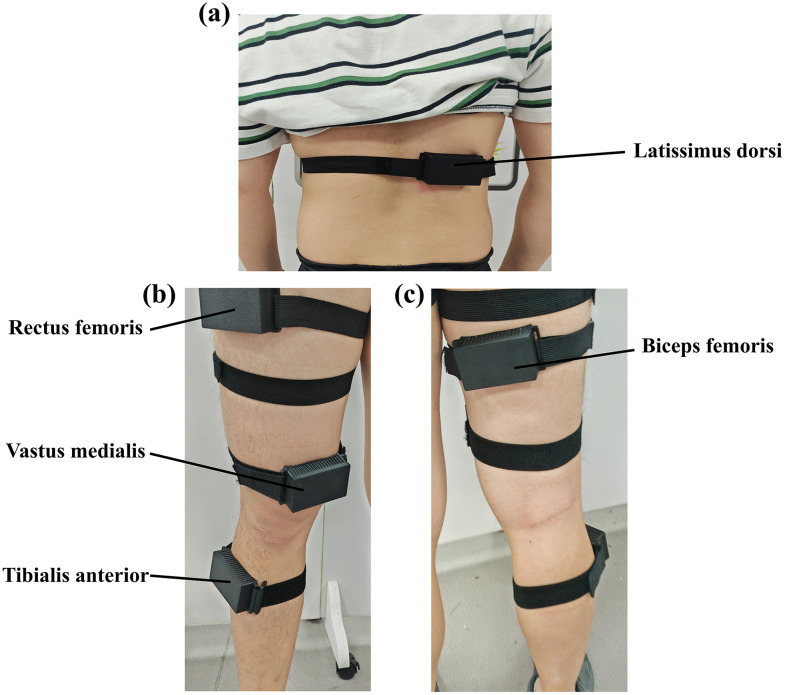
Sensor Placement Position. **(a)** Back (latissimus dorsi); **(b)** Anterior leg (rectus femoris, vastus medialis, tibialis anterior); **(c)** Posterior leg (biceps femoris).

For the evaluation of exoskeleton-assisted repetitive squat-lifting efficacy, five skeletal muscles were selected for MMG signal acquisition based on established research [33]—these muscles exhibit high activation levels during lifting movements, receive direct mechanical assistance from the exoskeleton, exert greater force during dynamic tasks, and their collective fatigue state reflects integrated bodily fatigue responses. The selected muscles were the rectus femoris, vastus medialis, biceps femoris, tibialis anterior, and latissimus dorsi, with detailed anatomical sensor placement presented in Fig 3.

MMG signals were collected using a custom wireless acquisition system based on an MPU9150 MEMS accelerometer. The MPU9150 MEMS accelerometer sensors were firmly fixed onto the skin surface of target muscles using high‑elasticity, non‑slip elastic straps, which maintained tight and stable sensor–skin contact throughout continuous dynamic squat‑lifting motions. Standardized sensor placement and consistent strap tension were applied across all participants to minimize inter‑subject variability in contact pressure and sensor positioning. No obvious sensor shifting, detachment, or signal loss was observed during any experimental trial. Analog outputs were transmitted via I2C to an STM32F411CCU6 microcontroller, which encoded the raw data into digital packets and wirelessly transmitted to a desktop receiver via an nRF24L01 (2.4 GHz) module. All signals were sampled at a 200 Hz sampling rate and stored in CSV format for offline processing.

**Fig 4:**
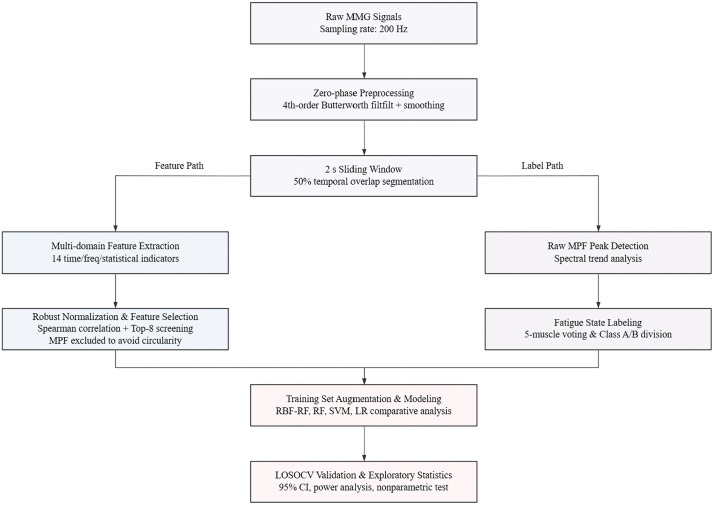
MMG Signal Processing Classification Flowchart.

The overall processing pipeline of MMG signals is illustrated in Fig 4. Raw MMG signals were first preprocessed to isolate physiological vibrations. A 4th-order zero-phase Butterworth bandpass filter with a passband of 5–50 Hz was applied to suppress low-frequency motion artifacts and high-frequency electronic noise; the filter was implemented using forward–reverse filtering (filtfilt) to avoid phase distortion. Following filtering, signal smoothing was performed using an 11-point moving average window to reduce high-frequency fluctuations. The filtered and smoothed signals were then converted to absolute values and segmented into sliding time windows of 2 s (400 samples at 200 Hz) with 50% temporal overlap (step length = 1s = 200 samples) for feature extraction.

Feature extraction was performed on each segmented window, generating 14 MMG features across three categories. Time-domain features included Mean Absolute Value (MAV), Root Mean Square (RMS), integrated MMG (iMMG), variance (VAR), waveform length (WL), slope sign changes (SSI), and MAV slope (MAVS). Frequency-domain features included Mean Frequency (MNF), Peak Frequency (PeakF), total spectral power (TotalPow), and central frequency (CF). Higher-order statistical features included skewness, kurtosis, and sample entropy (SampEn, embedding dimension m = 2, tolerance r = 0.15 × standard deviation of the segment). All extracted features were normalized per subject and per muscle using robust min–95th percentile scaling to reduce outlier influence. For each feature and muscle within a single subject, raw values were scaled to the range [0, 1] using: $x_{norm}=\frac{x_{raw}-x_{min}}{x_{\text{9}\text{5}th}-x_{min}+{\text{1}\text{0}}^{-\text{8}}}$, where $x_{min}$=minimum raw feature value, $x_{\text{9}\text{5}th}$= 95th percentile of raw feature values (used as upper bound to limit outlier impact). Fatigue state reference labels were defined based on raw Mean Power Frequency (MPF) temporal trends, a well-validated rule-based approach for muscle fatigue annotation in fused sEMG-MMG signal analysis [31]. No additional normalization was applied to MPF for label generation, preserving its original spectral unit. For each muscle, the 95th percentile of raw MPF values was used as a reference threshold for peak detection. A valid MPF peak was identified as a local maximum that exceeded 75% of this 95th-percentile reference and occurred at least 3 samples after window onset. Fatigue onset was defined as the first time point showing a sustained downward trend after this peak, defined by either: (1) two consecutive local minima; (2) three continuous declining samples; or (3) MPF falling below 88% of the peak value or 92% of the 95th-percentile reference. The pre-peak phase was labeled as Class A (non-fatigued), and the post-peak sustained decline phase was labeled as Class B (fatigued). Whole-body fatigue state was determined using a 5-muscle voting scheme. A window was classified as fatigued (Class B) when the number of individually fatigued muscles met or exceeded a predefined voting threshold. Four thresholds (1/5, 2/5, 3/5, 4/5) were tested for sensitivity analysis to evaluate the robustness of fatigue state definition.

### 2.4. Classification framework and validation

A classification framework was developed to identify whole-body fatigue states (Class A: non-fatigued; Class B: fatigued) based on multi-channel MMG features. Prior to model training, feature selection was performed to reduce redundancy and enhance robustness.

All candidate features were first evaluated using Spearman rank correlation (with two-tailed P-value) with interpolated subjective exertion (RPE) scores, together with variance analysis. Statistical significance was set at p < 0.05. Features were ranked by absolute Spearman correlation coefficient and variance, and the top eight statistically significant features were selected. MPF was explicitly excluded from the training feature set to mitigate potential circularity between feature representation and label definition.

The primary model was a hybrid Radial Basis Function–Random Forest (RBF–RF) classifier, combining nonlinear feature mapping with ensemble learning. An RBF kernel approximation (γ = 1.0, 100 components) was applied to project features into a higher-dimensional space, followed by a Random Forest classifier (150 trees, random seed = 42). To contextualize performance, three baseline models were implemented for comparison: standard Random Forest (RF), Support Vector Machine (SVM), and Logistic Regression (LR), all using default configurations without extensive hyperparameter tuning. This structured comparison of model variants and feature configurations follows the systematic ablation paradigm demonstrated in prior multimodal sensing classification work [34], which validates model design through component-wise and feature-set ablation.

To address the small sample size (*N* = 5) and reduce overfitting, lightweight feature-level data augmentation was applied exclusively to the training set within each cross-validation fold. Three augmentation operations were adopted to generate augmented samples: (1) additive Gaussian noise (σ = 0.01 × feature mean), (2) amplitude scaling (0.95–1.05), and (3) temporal shifting (±1 sample). Each original training sample generated three augmented copies, leading to a fourfold expansion of the training dataset. A controlled experiment comparing model performance with and without data augmentation was conducted to validate the effectiveness of the augmentation strategy.

Model evaluation was conducted using Leave-One-Subject-Out Cross-Validation (LOSOCV), where each subject was iteratively used as an independent test set while the remaining subjects formed the training set. This approach ensures strict separation between training and testing data and evaluates cross-subject generalization.

To assess the robustness of fatigue state definition, four multi-muscle voting thresholds (1/5, 2/5, 3/5, and 4/5) were evaluated. Model performance was assessed using accuracy, Area Under the Receiver Operating Characteristic Curve (AUC), F1-score, and specificity. A temporal metric, defined as the absolute error between predicted and reference fatigue onset time, was also computed. In addition, Spearman’s correlation coefficient between predicted fatigue states and subjective RPE scores was used to evaluate convergent validity. To quantify inter-subject variability, results were reported as mean ± standard deviation (SD), along with 95% confidence intervals (CI) estimated using the t-distribution. Post hoc power analysis was performed to calculate the effect size (Cohen’s d) and statistical power (1 − β) for the primary metric of fatigue onset time error, verifying the statistical reliability of the experimental results.

### 2.5. Outcome measures and statistical analysis

Given the pilot nature of the study and the limited sample size (*N* = 5), all statistical analyses were explicitly framed as exploratory, and inferential statistics are reported descriptively to indicate trends rather than to support confirmatory hypothesis testing. Primary and secondary outcome measures included Fatigue Onset Latency (duration from trial start to the first sustained model-predicted Class B state) and Subjective-Objective Alignment (Spearman’s rank correlation between model-predicted states and participant-reported subjective exertion scores to assess convergent validity).

To quantify uncertainty and inter-subject variability induced by the small sample, 95% confidence intervals (CI) were additionally computed using the Student’s t-distribution for all key metrics. For machine learning model evaluation, subject-wise performance distributions were systematically quantified under LOSOCV, including accuracy, AUC, F1-score, specificity, and fatigue onset time error.

Descriptive statistics (mean ± standard deviation, SD) described central tendency and variability; normality of time to fatigue onset was assessed via the Shapiro-Wilk test (α = 0.05). Paired-samples t-tests or nonparametric Wilcoxon signed-rank tests compared outcomes between assisted and unassisted conditions. Spearman’s rank correlation evaluated monotonic relationships between objective states and ordinal subjective exertion scores.

For machine learning performance assessment, metrics were averaged across LOSOCV folds and reported with mean ± SD and 95% CI to reflect small-sample uncertainty. The performance comparison between augmented and non-augmented training data was reported using the same statistical metrics to validate the efficacy of data augmentation. Post hoc power analysis was integrated to quantify the statistical adequacy of the small-sample experiment, with results reported as effect size and statistical power. Statistical significance was set at α = 0.05.

## 3. Results

### 3.1. Integrated analysis of feature selection and model performance

Feature importance was quantified using Spearman correlation with RPE and feature variance (Table 2). Most features showed statistically significant but weak associations (|ρ| < 0.35, p < 0.05; 10 features: p < 0.001), while PeakF and SampEn exhibited no significant correlation (p > 0.05). MAV and iMMG achieved the highest correlation coefficients, followed by RMS, SSI, WL, MAVS, VAR, and TotalPow. The top 8 statistically significant features were selected for subsequent model training.

**Table 2:** Correlation and significance statistics of the features with RPE.

Feature	Spearman ρ	P-value	Variance
**MAV**	0.2106	0.0001	0.1042
**iMMG**	0.2106	0.0001	0.1042
**RMS**	0.1720	0.0001	0.1077
**SSI**	0.1625	0.0001	0.1127
**WL**	0.1535	0.0001	0.0981
**MAVS**	0.1535	0.0001	0.0981
**VAR**	0.1344	0.0001	0.1742
**TotalPow**	0.1265	0.0001	0.2457
**Kurtosis**	0.1027	0.0001	0.1151
**Skewness**	0.0978	0.0001	0.0879
**MNF**	0.0495	0.0497	0.0791
**CF**	0.0495	0.0497	0.0791
**PeakF**	0.0322	0.2026	0.3781
**SampEn**	0.0295	0.2433	0.0751

Four classification models (RBF-RF, RF, SVM, Logistic Regression) were used for performance comparison under all feature combinations and voting rules. The top ten highest classification accuracies across all combinations were achieved by the RBF-RF model. Among all feature-model-voting rule combinations, the optimal combination of Logistic Regression ranked 16th, that of RF ranked 274th, and that of SVM ranked 412th. Additionally, 76% of the top 100 combinations were obtained by the RBF-RF model. The optimal combination and corresponding performance indicators of each model are listed in Table 3.

**Table 3:** Performance comparison of four classifiers.

Model	Feature Combination	Acc Mean±SD (95% CI)	AUC Mean±SD (95% CI)	F1 Mean±SD	Spe Mean±SD	Fatigue Onset Time Error Mean±SD
**RBF**-**RF**	MAV-iMMG-RMS-WL-TotalPow	0.9134 ± 0.057 (0.8343,0.9925)	0.7922 ± 0.0729 (0.6910, 0.8933)	0.9482 ± 0.036	0.5844 ± 0.1457	1.4 ± 0.80
**Logistic Regression**	RMS	0.9073 ± 0.0562 (0.8292,0.9853)	0.7745 ± 0.0706 (0.6765, 0.8725)	0.9448 ± 0.0355	0.549 ± 0.1413	1.4 ± 0.80
**RF**	RMS-SSI-VAR	0.8967 ± 0.0435 (0.8363,0.9571)	0.7972 ± 0.07 (0.7001, 0.8944)	0.9371 ± 0.0288	0.6273 ± 0.1491	1.4 ± 0.80
**SVM**	WL-MAVS	0.8904 ± 0.0294 (0.8496,0.9312)	0.8233 ± 0.0662 (0.7315, 0.9151)	0.9324 ± 0.0195	0.7045 ± 0.1531	1.2 ± 0.98

### 3.2. Sensitivity analysis of voting rules

Sensitivity analysis was performed on four voting rules (1/5, 2/5, 3/5, 4/5). For each rule, all feature combinations and four classification models were traversed to obtain the optimal configuration.

**Fig 5:**
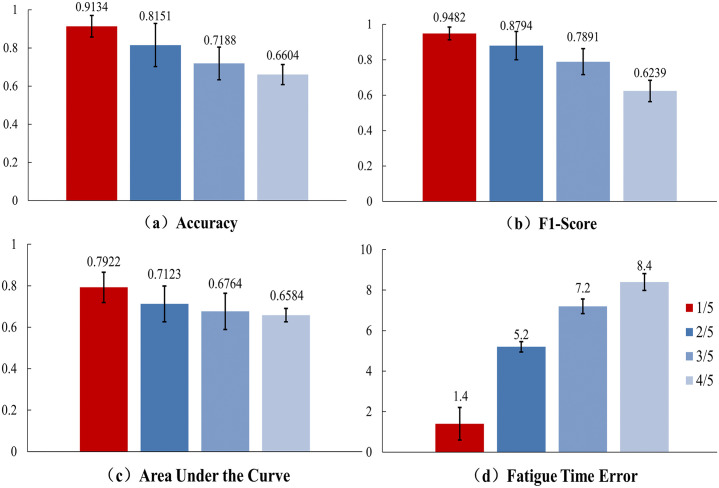
Sensitivity analysis of voting rules. **(a)** Mean classification accuracy, **(b)** mean F1-score, **(c)** mean AUC, and **(d)** mean fatigue onset time error across four voting rules (1/5, 2/5, 3/5, 4/5).

As shown in Fig 5 and Table 4, classification performance (accuracy, F1-score, AUC) decreased monotonically as the voting threshold became stricter, while fatigue onset time error increased. The 1/5 voting rule yielded the best overall performance, with mean accuracy of 0.9134 ± 0.0570, mean AUC of 0.7922 ± 0.0729, mean F1-score of 0.9482 ± 0.0360, and the lowest mean fatigue onset time error of 1.4 ± 0.8 s.

**Table 4:** Performance under different voting rules.

Voting Rule	Mean Accuracy ± SD (95% CI)	Mean AUC ± SD(95% CI)	Mean F1-Score ± SD	Mean Fatigue Time Error ± SD (s)
**1/5**	0.9134 ± 0.0570 (0.8343, 0.9925)	0.7922 ± 0.0729 (0.691, 0.8933)	0.9482 ± 0.0360	1.4 ± 0.8
**2/5**	0.8151 ± 0.1127 (0.6586, 0.9716)	0.7123 ± 0.0860 (0.574, 0.8798)	0.8794 ± 0.0799	5.2 ± 2.79
**3/5**	0.7188 ± 0.0855 (0.6002, 0.8375)	0.6764 ± 0.0869 (0.5557, 0.7971)	0.7891 ± 0.0731	7.2 ± 3.49
**4/5**	0.6604 ± 0.0525 (0.5875, 0.7333)	0.6584 ± 0.0322 (0.6137, 0.7031)	0.6239 ± 0.0601	8.4 ± 4.5

### 3.3. Subject-wise performance analysis

Under the optimal model combination, the subject-wise performance indicators for muscle fatigue prediction under exoskeleton wearing condition are shown in Table 5. The optimal model adopted 1/5 voting rule, combined with MAV-iMMG-RMS-WL-TotalPow/ MAV-iMMG-RMS-MAVS-TotalPow feature set and RBF_RF classifier. Across all 5 subjects, the prediction accuracy ranged from 80.95% to 96.55%, with a mean accuracy of 91.34%. The F1-score varied between 0.8824 and 0.9804 (mean = 0.9482), and the AUC ranged from 0.6667 to 0.8750 (mean = 0.7922). The mean prediction time error of fatigue onset was 1.40 ± 0.89 s, and the mean Spearman correlation between predicted strain state and subjective RPE was 0.4818.

**Table 5:** Subject-wise prediction performance under optimal model.

Subject	Accuracy	AUC	F1 Score	Specificity	Time Error (s)	Spearman Correlation
**DQ01**	0.8095	0.6667	0.8824	0.3333	1.00	0.5139
**DQ02**	0.9583	0.8500	0.9764	0.7000	1.00	0.4151
**DQ03**	0.9355	0.8000	0.9630	0.6000	3.00	0.3922
**DQ04**	0.9655	0.8750	0.9804	0.7500	1.00	0.5318
**DQ05**	0.8983	0.7692	0.9388	0.5385	1.00	0.5550

Post hoc power analysis was performed using the fatigue onset time error derived from the optimal model. The statistical results are as follows in Table 6. The Cohen’s d effect size was 1.565. The statistical power (1 − β) of one-sample t-test was 0.745, and that of independent-samples t-test was 0.585 at α = 0.05.

**Table 6:** Post-hoc power analysis results.

Statistical Item	Value
**Time error of fatigue onset (Mean±SD, s)**	1.40 ± 0.89
**Cohen’s d**	1.565
**Power of one-sample t-test (1 − β)**	0.745
**Power of independent-samples t-test (1 − β)**	0.585

### 3.4. Data augmentation and statistical power analysis

The comparison between data augmentation and non-data augmentation was performed using the globally optimal configuration, which was determined by traversing 4 voting rules, all feature combinations under each voting rule, and 4 models under each feature combination to select the combination with the highest overall accuracy.

With data augmentation, the mean accuracy improved from 0.9039 ± 0.0581 to 0.9134 ± 0.0570 (1.05% improvement), the mean AUC increased from 0.7723 ± 0.0721 to 0.7922 ± 0.0729 (2.58% improvement), and the mean F1-score rose from 0.9426 ± 0.0367 to 0.9482 ± 0.0360 (0.59% improvement). The mean fatigue onset time error remained unchanged at 1.4 ± 0.8 s under both conditions (Table 7).

**Table 7:** Performance comparison with and without data augmentation.

Indicator	With Data Augmentation	Without Data Augmentation
**Mean Accuracy ± SD**	0.9134 ± 0.057	0.9039 ± 0.0581
**Mean AUC ± SD**	0.7922 ± 0.0729	0.7723 ± 0.0721
**Mean F1-Score ± SD**	0.9482 ± 0.036	0.9426 ± 0.0367
**Mean Fatigue Time Error ± SD**	1.4 ± 0.8	1.4 ± 0.8

### 3.5. Physiological validation

To validate the proposed MMG-based monitoring framework, the fatigue states predicted by the optimal model (RBF-RF with a 1/5 voting rule) was evaluated against subjective exertion.

**Fig 6:**
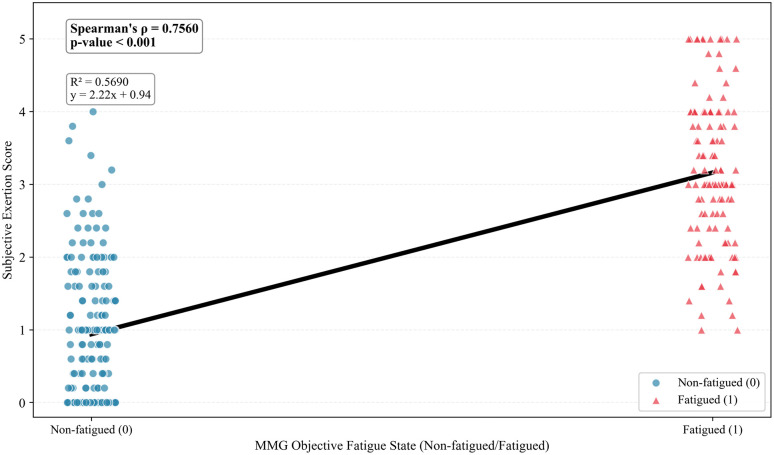
Correlation of Objective MMG Fatigue and Subjective Exertion.

Spearman’s rank correlation analysis revealed a significant monotonic association between the predicted fatigue states and subjective exertion scores (ρ = 0.7560, p < 0.001). Linear regression further demonstrated a moderate relationship (R² = 0.5690), described by the model: Subjective Exertion Score = 2.22 × Fatigue State + 0.94 (Fig 6).

## 4. Discussion

### 4.1. Feature characterization and classification performance

All extracted MMG features exhibit weak-to-moderate associations with subjective exertion (∣ρ∣ < 0.35), which suggests that no single feature is sufficient to fully characterize muscle fatigue during dynamic squat-lifting. Among the evaluated indicators, time-domain features such as MAV and iMMG show relatively higher correlations, followed by RMS and other derived metrics. This observation is consistent with the inherently non-stationary nature of MMG signals during dynamic contractions [35]. Unlike isometric conditions, continuous changes in muscle length, joint motion, and sensor displacement likely introduce substantial variability into frequency-domain features [36]. Consequently, individual spectral indicators appear more susceptible to motion artifacts and local signal fluctuations. In contrast, time-domain features, such as MAV and iMMG, may provide a more stable quantification of mechanical activity and motor unit recruitment intensity throughout the movement.

A systematic performance evaluation was implemented across four classification frameworks: RBF-RF, conventional RF, SVM, and Logistic Regression. Over 4,080 integrated model-feature-rule configurations were screened. The top ten configurations, ranked by classification accuracy, were entirely dominated by the RBF-RF framework. Furthermore, RBF-RF accounted for 76% of the top 100 high-performance schemes, while the optimal configurations of Logistic Regression, RF, and SVM ranked 16th, 274th, and 412th, respectively.

As summarized in Table 3, the RBF–RF model utilizing the MAV–iMMG–RMS–WL–TotalPow feature combination yielded the highest mean accuracy of 0.9134 ± 0.057 (95% CI: 0.8343, 0.9925) and an optimal F1-score, with a narrow 95% CI range reflecting more stable predictive performance. Logistic Regression delivered comparable accuracy and F1 metrics, while RF exhibited balanced AUC and specificity. SVM, though achieving the highest specificity (Spe), showed relatively lower classification accuracy, and minor differences in fatigue onset time error were observed across all four models. While Logistic Regression delivered comparable performance metrics, the RBF–RF framework exhibited a tendency toward better performance. This performance may benefit from the combination of RBF nonlinear mapping and random forest ensemble learning. RBF transformation helps capture nonlinear patterns in MMG signals, while the ensemble structure reduces overfitting risks in small-sample settings [37]. Compared with SVM, RBF-RF shows better noise robustness, which aligns with the observation that random forests are more stable under noisy conditions than SVM [38].

It is important to acknowledge that these findings are preliminary, given the limited sample size (*N* = 5) and inherent variability in human movement. These results indicate that fatigue-related information is distributed across multiple complementary MMG features rather than dominated by a single indicator, supporting the advantage of a multi-feature fusion approach for constructing a robust fatigue evaluation model instead of relying solely on an individual physiological parameter. Additionally, the observed superiority of the RBF–RF framework should be regarded as an exploratory indication rather than a definitive conclusion; further validation with a larger, more diverse cohort is essential to confirm its generalizability, long-term monitoring stability, and clinical utility for MMG-based muscle fatigue assessment.

### 4.2. Sensitivity analysis and implications of voting rules

Sensitivity analysis of four voting thresholds (1/5, 2/5, 3/5, 4/5) revealed a monotonic trend: stricter voting rules led to gradual declines in comprehensive classification metrics including accuracy, F1-score and AUC, alongside a marked increase in fatigue onset time error. Within this small-sample pilot investigation, the 1/5 threshold delivered the optimal overall performance, presenting the highest mean accuracy (0.9134 ± 0.0570), F1-score (0.9482 ± 0.0360) and AUC (0.7922 ± 0.0729), as well as the minimum temporal error of fatigue detection (1.4 ± 0.8 s). In contrast, the strictest 4/5 rule produced the lowest classification accuracy (0.6604 ± 0.0525) and the longest detection delay (8.4 ± 4.5 s), as shown in Fig 5. and Table 4.

This observation aligns with the fundamental physiological nature of muscle fatigue during multi-joint dynamic movements. As established by Enoka and Duchateau [39], muscle fatigue does not develop uniformly across a muscle group; instead, it manifests heterogeneously, with onset varying between muscles based on their functional role, load distribution, and fiber-type composition. For multi-muscle dynamic tasks (e.g., the five-muscle system in this study), fatigue first manifests in lead muscles that bear disproportionate mechanical demand, long before a global state of muscular exhaustion is reached [40]. Notably, the superior performance of the 1/5 voting scheme may reflect early localized fatigue detection rather than a confirmed physiological mechanism.

Stringent majority-based voting strategies have been adopted in previous physiological fatigue monitoring [31], especially for industrial scenarios like manual lifting, where high specificity and low false-alarm rates are prioritized to guarantee operational safety. Nevertheless, these conservative criteria may be poorly suited for wearable exoskeleton systems, which rely on low-latency feedback to deliver proactive biomechanical assistance. The strict rule-induced delay would render exoskeleton support passive rather than preventive, compromising its core assistive function. Comparatively, the permissive 1/5 voting threshold may enable more timely fatigue detection, and its advantages in temporal responsiveness outweigh the potential risk of false positives under controlled laboratory conditions.

Several limitations should be noted when interpreting the current findings. Restricted by the small sample size (n = 5), the superiority of the 1/5 rule is only validated for the specific dynamic exoskeleton task and tested cohort, and cannot be generalized to larger populations or alternative movement paradigms. Though false-positive events were rarely observed in controlled laboratory environments, the feasibility of the loose 1/5 threshold still requires further field validation to confirm long-term stability in noisy, real-world conditions. Overall, the selection of muscle fatigue voting criteria is highly task-dependent. The lenient 1/5 scheme may be more applicable to time-critical exoskeleton assistance requiring early fatigue detection, while stricter voting thresholds remain indispensable for industrial tasks that demand conservative fatigue judgment and high specificity.

### 4.3. Subject-wise performance and statistical power under small sample size

Conducting this study on a small cohort (N = 5) is a standard practice for pilot-scale methodological validations in MMG research [41]. The observed Cohen’s d of 1.565 for fatigue onset error represents a “very large” effect size, suggests that the MMG-based framework captures a robust physiological signal. However, the post-hoc power analysis revealed a discrepancy between the one-sample t-test (Power = 0.745) and independent-samples comparisons (Power = 0.585). The lower power for between-condition comparisons (Exo vs. No-Exo) suggests that the current sample size is insufficient to overcome the high inter-subject variability inherent in human-robot interaction. This variability is consistent with prior reports that MMG-based fatigue detection exhibits low cross-subject consistency and requires subject-specific training, and that neuromuscular force control and fatigue responses are highly individualized due to distinct motor unit recruitment and muscle properties [42].This variability implies that a “one-size-fits-all” model may be suboptimal. For future exoskeleton applications, these results support the adoption of “personalized calibration” protocols—where the model is fine-tuned using a brief initial session for each new user—rather than relying solely on large-scale, generalized datasets.

### 4.4. Role of data augmentation in enhancing model robustness

Data augmentation provided modest but consistent improvements in classification performance, with gains in accuracy (1.05%), AUC (2.58%), and F1-score (0.59%), while fatigue onset time error remained unchanged. In the context of a pilot study with limited data, a conservative augmentation strategy (minor temporal shifts, amplitude scaling, and noise perturbation) was adopted to avoid introducing physiologically implausible artifacts [43]. The improvement in AUC is particularly meaningful: it demonstrates that the model learned more stable decision boundaries invariant to typical wearable sensor disturbances, such as electrode micro-displacements and signal amplitude fluctuations [44].

While data augmentation cannot replace a larger dataset, it acts as a critical regularizer for physiological monitoring applications where data collection is physically taxing for participants. Even mild, realistic augmentation can effectively boost model generalization without distorting the underlying physiological signal [45].

### 4.5. Physiological validation of the MMG-based monitoring framework

The association was identified between the fatigue states predicted by the RBF-RF framework and subjective ratings of perceived exertion (RPE) (ρ = 0.7560, p < 0.001), providing preliminary evidence supporting the physiological relevance of the MMG-based fatigue monitoring approach in this small sample (*N* = 5). Notably, this higher correlation was computed on pooled overall data across all participants, whereas the relatively lower subject-wise correlation values summarized in Table 5 are largely driven by inherent inter-subject neuromuscular variability. The imperfect correlation observed here reflects well-documented theoretical differences between central perceptual experience and peripheral physiological fatigue: RPE constitutes a global subjective construct that integrates cardiovascular, psychological, and multisensory afferent inputs [46], while MMG specifically quantifies the peripheral mechanical activity of skeletal muscle motor units. As established by Marcora [47], perception of effort during movement arises primarily from central motor command signals rather than direct reliance on peripheral afferent feedback, which accounts for the divergence between the central subjective sense of exertion and the localized peripheral fatigue captured by MMG.

### 4.6. Limitations and future work

Several limitations should be acknowledged. Following the paradigm of sensor-based human motor analysis [48], this study focuses on validating an MMG-derived fatigue proxy rather than directly evaluating exoskeleton efficacy.

First, fatigue labels were defined solely by MPF-based rules. Although MPF was excluded from model training to avoid circular dependency, no independent physiological gold standards (e.g., sEMG median frequency, torque decay, metabolic cost) were used for cross-validation, leaving the labels’ physiological validity unconfirmed. Additionally, concurrent biomechanical/metabolic measurements (sEMG, torque, metabolic cost) were lacking to externally validate predicted fatigue states. Thus, we cannot determine whether model outputs reflect actual functional fatigue (e.g., force loss) or merely MMG signal characteristics. Second, the small sample size (N = 5) limits statistical power and generalizability. Third, fixed laboratory conditions (10 kg load, metronome-paced speed) restrict ecological validity; performance under variable loads, speeds, and unstructured environments remains untested. Motion artifacts and interference in field conditions, as well as the anti-interference capability of our MMG processing pipeline, have not been validated. Moreover, limited by the small sample, we only used equal-weight multi-muscle voting without exploring weighted voting based on muscle activation intensity.

Future work should prioritize independent physiological gold standards to validate MMG-derived labels, consider multi-modal fusion (MMG + sEMG), test performance under variable loads/speeds, assess motion artifacts in real-world scenarios while optimizing preprocessing to suppress interference, conduct large-cohort and field validation, explore weighted voting based on muscle activation intensity, and develop subject-specific personalized models.

## 5. Conclusion

This pilot study investigated the feasibility of monitoring neuromuscular fatigue using MMG signals combined with machine learning during repetitive squat-lifting tasks in a small cohort (*N* = 5). The results suggest that fatigue-related neuromuscular characteristics are reflected across multiple complementary MMG features, supporting the use of multi-feature fusion rather than reliance on a single physiological indicator. A conservative data augmentation strategy resulted in modest but consistent improvements in classification performance, including increases in accuracy, AUC, and F1-score, and may contribute to enhanced robustness against common wearable sensor disturbances. This finding indicates that physiologically plausible augmentation can serve as a useful regularization approach when working with limited datasets. Among the evaluated configurations, the RBF–RF model combined with a 1/5 voting rule achieved comparatively strong overall performance and demonstrated sensitivity to early fatigue-related changes. In addition, a significant association between model-predicted fatigue states and subjective perceived exertion was observed, providing preliminary evidence supporting the physiological relevance of the proposed framework. Given the small sample size and controlled experimental setting, these findings should be interpreted with caution.

Overall, this exploratory study provides initial evidence for the feasibility of MMG-based fatigue monitoring in wearable assistive systems. Future work involving larger cohorts, multi-scenario validation, and real-world testing is necessary to further evaluate the generalizability and practical applicability of the proposed approach.
